# Evaluating the OH-EpiCap tool using the Danish integrated surveillance program for AMU and AMR as a case study

**DOI:** 10.3389/fpubh.2023.1127701

**Published:** 2023-11-20

**Authors:** Pedro Moura, Birgitte Borck Høg, Lis Alban, Ute Wolff Sönksen, Ana Sofia Ribeiro Duarte, Marianne Sandberg

**Affiliations:** ^1^National Food Institute, Technical University of Denmark, Lyngby, Denmark; ^2^Department for Food Safety, Veterinary Issues and Risk Analysis, Danish Agriculture and Food Council, Copenhagen, Denmark; ^3^Department of Veterinary and Animal Sciences, University of Copenhagen, Frederiksberg, Denmark; ^4^Statens Serum Institut, Copenhagen, Denmark

**Keywords:** One Health, surveillance, antimicrobial, resistance, consumption, stewardship

## Abstract

Antimicrobial resistance (AMR) is considered a One Health (OH) challenge, ideally demanding concerted efforts from the animal, human and environmental side. DANMAP, the Danish Integrated Antimicrobial Resistance Monitoring and Research Program, is monitoring AMR and antimicrobial use in animals and humans. OH-EpiCap is an evaluation tool, developed to address essential elements in OH surveillance systems, such as the dimensions of the organization, operational activities and the impact of the surveillance activities. We aimed to evaluate DANMAP using OH-EpiCap and hereby assessed the suitability of OH-EpiCap to evaluate integrated AMR surveillance systems. During the evaluation, the strengths and weaknesses of DANMAP concerning the “OH-ness” of the program were discussed. Furthermore, possible adaptations of the standard operating procedures and governance structure were addressed. Attention was paid to the ability and easiness of DANMAP to cope with current and future challenges connected to integrated AMR surveillance. It was concluded that DANMAP has a strong OH approach covering relevant aspects for humans and animals, whereas environmental aspects are missing. OH-EpiCap proved to be straightforward to use and provided valuable insights. The authors recommend OH-EpiCap to be used by health authorities and stakeholders. It is not suitable for the technical evaluation of a surveillance program.

## Introduction

Antimicrobial resistance (AMR) has been defined a cross-sectoral problem due to it affecting both animals and humans, carrying an inbound risk of circulation within and between both domains. In addition, the environment can serve as a “melting pot” for both antimicrobial resistant bacteria and genes (1). The exchange among populations often happens sporadically, but AMR may accumulate over time, and at the time of detection, the root causes are not always easy to establish (2–4). Nonetheless, the antimicrobial use (AMU) in one sector may contribute to the development of resistance in another sector, as it has been demonstrated by both scientific publications (5–7) and the joint report on antimicrobial usage and antimicrobial resistance in humans and food producing animals in the European Union (8).

Integrated surveillance programs are based on multi-sectoral collaborative activities such as the collection, analysis and dissemination of the results to relevant stakeholders across sectors for action and policymaking (9). In AMR surveillance programs, multi-level integration is considered crucial to untangle the consequences related to AMR. Connecting the political decision level to the technical surveillance activity level is essential to sustain risk mitigation decisions against the defined health hazards (10).

In concordance with what is described above, the One Health High-Level Expert Panel (OHHLEP) of the World Health Organization (WHO) defines One Health (OH) as “an integrated, unifying approach that aims to sustainably balance and optimize the health of people, animals, and ecosystems,” recognizing that they are closely linked and interdependent (11). The OHHLEP supports the described horizontal, e.g., cross-sectoral, and vertical, multi-level, approach to integrated surveillance systems and encourages the upscaling of intersectoral collaboration in national strategies against AMR by use of the strategic framework developed by the Panel (12).

The Danish program for surveillance of AMU and AMR in bacteria from food animals, food, and humans (DANMAP) was established in 1995. Collaboration and cross-sectoral decision-making have always served as a fundament of the program (13). The objectives of DANMAP are: (i) to monitor AMU in food animals and humans, as well as the occurrence of AMR in bacteria isolated from food animals, food of animal origin and humans; (ii) to study associations between AMU and AMR; (iii) to identify routes of transmission and areas for further research.

The ultimate objective of the program is to produce information that sustains risk mitigation actions connected to AMR hazards that might affect humans and/or animals. Several bacterial hazards with different types of AMR are under surveillance, i.e., bacteria isolated from patients, zoonotic bacterial pathogens (*Salmonella* spp. and *Campylobacter* spp.), and indicator and pathogenic bacteria from food producing animals (pigs, cattle and broilers) and from food products thereof (13).

According to Aenishaenslin et al. (9), the added value of integration in AMR surveillance can be projected or recognized in the system’s performance in different outcomes: (1) immediate outcomes as increased epidemiological knowledge from the combination of collected data; (2) intermediate outcomes, by causing behavior and policy changes that can be reflected in AMR trends; (3) ultimate outcomes such as reductions in overall AMU and AMR, leading to measurable improvements of health in the affected domains (9). In line with Aenishaenslin et al. (9), the epidemiological knowledge generated by the DANMAP program itself and associated research have contributed significantly to actions in the Danish livestock farming industry, such as the voluntary ban on the use of third- and fourth-generation cephalosporins in the Danish pig industry (14), which ultimately led to these substances not being used at all in Danish pig production (13).

In 2017, a new Danish National OH Strategy Against Antibiotic Resistance was issued by the Ministry of Food, Agriculture and Environment together with the Ministry of Health, reiterating existing initiatives on AMR prevention, mitigation and control (15). In Denmark, due to the large size of the pig sector compared to the size of the country, the AMU in pigs has been in focus for decades. This has resulted in a relatively low AMU per pig as shown by Moura et al. (16). The many actions implemented in Denmark to guide AM stewardship can be consulted in the latest DANMAP report (13).

Scientific and technological advancements together with emerging or potentially threatening health challenges can justify changes in a surveillance program. Therefore, it is important to evaluate a program’s performance in meeting the defined objectives, while operating under a set budget (17).

Given the sheer complexity of designing and operating a multi-sectorial national-scale program, the aim of evaluating the One Health-ness” (OH-ness) of DANMAP has been recognized as relevant by the program’s steering committee. As mentioned above, integration and collaboration are essential parts of OH. However, it is possible that increasing the level of integration and collaboration in all components of AMR surveillance would neither add value to the information generated nor improve decision-making. Therefore, the cost-effectiveness of changes in the integration and collaboration should be understood and evaluated critically (18).

The MATRIX consortium, funded by the One Health European Joint Program, developed the OH-EpiCap tool to systematically characterize epidemiological surveillance activities in a national surveillance system. The main aim of OH-EpiCap is to facilitate the assessment and improvement of national capacities and capabilities for integrated surveillance of zoonotic hazards (19). So far, due its generic design, the OH-EpiCap tool has been successfully applied to surveillance activities connected with food-borne hazards, such as *Salmonella*, *Campylobacter, Listeria* and other zoonotic hazards such as *Chlamydia psittaci*, however, it has so far not been used to evaluate AMR surveillance activities.

To provide guidance in choosing among evaluation tools for AMU and AMR surveillance systems, an international network called CoEvalAMR was established in 2019 (20). The CoEvalAMR network has recently evaluated nine AMR surveillance systems using OH-EpiCap (21). This case study is part of the aforementioned work. With the main objective of determining whether OH-EpiCap is suitable to evaluate a complex integrated AMR program, we evaluated DANMAP using OH-EpiCap. The outcomes of this evaluation serve as the basis for the secondary aim of this work, which was to present and briefly discuss the strengths and weaknesses of DANMAP in what concerns the OH-ness of the program.

## Materials and methods

### DANMAP

As proposed by the OH-EpiCap tool, a surveillance system can be decomposed into the following activities: (1) planning and management, (2) data collection, analysis and interpretation, and (3) distribution of the information generated (22).

**Figure 1:**
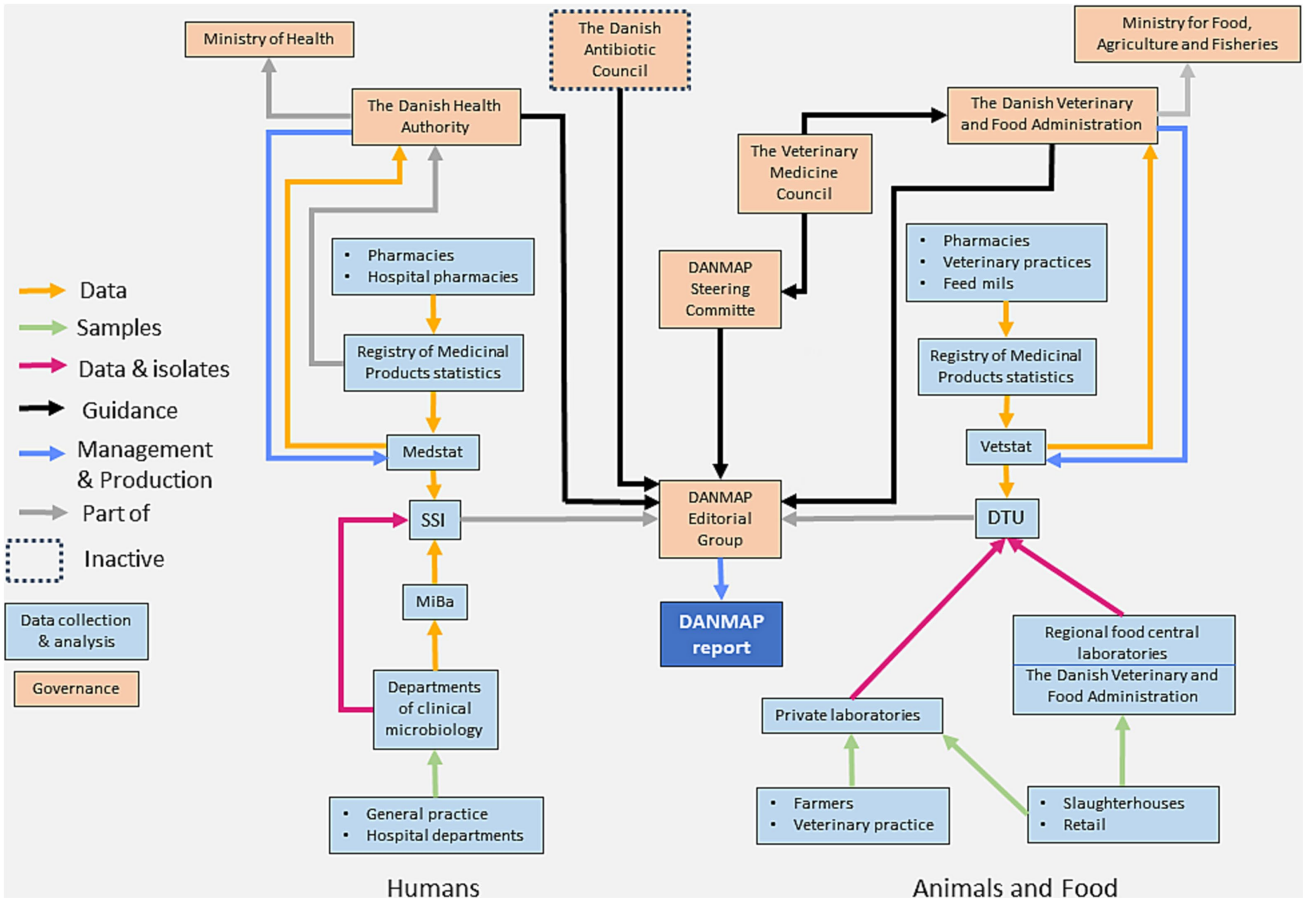
Organigram showing the governance structure and activity flow in DANMAP, as it was in 2022.

The governance structure and main activities that compose DANMAP are presented in Figure 1. DANMAP is managed by a collaborating team from the National Food Institute at the Danish Technical University (DTU) (EU Reference Laboratory for antimicrobial resistance) and the National Reference Laboratory for Antimicrobial Resistance at Statens Serum Institut (SSI). The program and its overarching goals are set by order of the Ministry of the Interior and Health of Denmark and the Ministry of Food, Agriculture and Fisheries of Denmark and financed via state funds. The Danish Antibiotic Council, currently not in function, was instructed to oversee the program. This task is now the responsibility of the two ministries and the Danish parliament. The governance structure of the program aims to ensure that all relevant parties can express their science-based advises and/or political views on the program’s design and on the implementation of activities. Currently, the Danish Veterinary Medicine Council (23), which is composed of experts from the animal and human side, provides advise to the Danish authorities and to DANMAP. In addition, DANMAP receives input and recommendations to guide the program’s priorities by multiple stakeholders with expertise in animal and public health. The stakeholders are the Danish Veterinary Association and the Danish Medical Association, the livestock producers, as well as other farmers’ organizations, animal health organizations, food and drug regulators and researchers. These stakeholders are invited to the annual stakeholder meeting, at which the results of the surveillance activity to be released in the yearly DANMAP report are presented.

DANMAP has no formalized evaluation methodology apart from regular technical reviews of data inclusion, quality and flow. After receiving scientific guidance from several parties, the steering committee, composed by representatives from DTU Food and SSI, coordinates, describes and prioritizes the program’s activities. The Danish Veterinary and Food Administration and the Danish Health Authority are the risk managers of the Danish AMR activities. Based on results from DANMAP, these agencies ultimately define and decide the priorities for the different initiatives and actions, such as, e.g., adjusting the permit limits that form part of the yellow card system for reduction of AMU in the pig sector and updating the guidelines for the management and control of highly resistant pathogens in the Danish healthcare system (24, 25).

In our evaluation, we assessed the AMU and AMR surveillance components of DANMAP, as a whole, whereas the management/execution part was only evaluated for the animal sector (Target 1.1 Formalization, as seen in Table 1).

**Table 1:** Dimensions and targets, each composed of four questions, evaluated by the OH-EpiCap—adapted after Tegegne et al. (19).

**Dimension 1: organization**			
Target 1.1 Formalization	Target 1.2 Coverage	Target 1.3 Resources	Target 1.4 Evaluation and resilience
**Dimension 2: operational activities**			
Target 2.1 Data collection and methods sharing	Target 2.2 Data sharing	Target 2.3 Data analysis and interpretation	Target 2.4 Communication
**Dimension 3: impact**			
Target 3.1 Technical outputs	Target 3.2 Collaborative added value	Target 3.3 Immediate and intermediate outcomes	Target 3.4 Ultimate outcomes

### The OH-EpiCap tool

**Figure 2:**
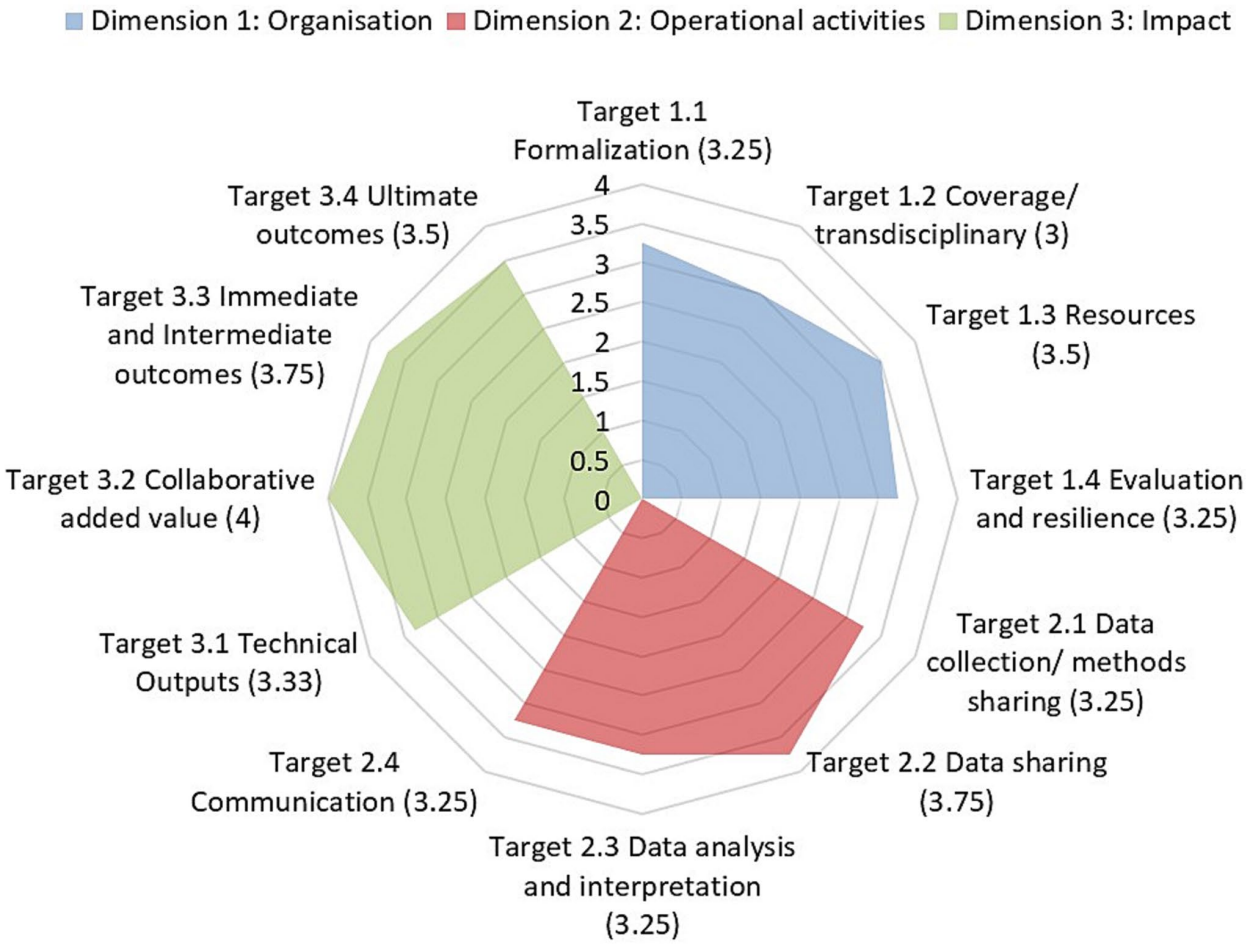
Average scores of DANMAP for the target areas covered by the OH-EpiCap tool, segmented into three dimensions. The average scores for each of the target areas are indicated in brackets. The figure was adapted from the report generated by the OH-EpiCap.

The OH-EpiCap tool is composed of three thematic domains/dimensions, each with four targets. These are further segmented into four questions, leading to a total of 48 standardized questions, which are used as indicators (Table 1). The 48 questions are answered using a semi-quantitative scale, ranging from 1 to 4, with 4 representing the best scenario regarding integrated OH surveillance. Respondents need to be familiar with the surveillance program of interest to answer the questions. The OH-EpiCap tool also comprises a dashboard, in which components are reported and where the average scores per target are presented in a radar diagram like the one presented in Figure 2.

### Application of the OH-EpiCap tool

The OH-EpiCap tool was last used by the authors on 18th of August 2022. Hence, the discussion reflects the questions as they were formulated and included in the OH-EpiCap version applied and the standard operating procedures of DANMAP at that time. The evaluation was conducted in two rounds, where the OH-EpiCap evaluation scheme was applied and discussed between representatives from the DANMAP management (*n* = 2), academia (*n* = 2), and the Danish livestock industry (*n* = 1). The assessors who formed part of the group provided answers based upon their work experience and views on the components of the system, Subsequently, the answers were discussed in the group and the final scores were obtained by consensus. Overall, when answering the individual questions that form part of OH-EpiCap, a conservative approach was chosen. Hence, when in doubt between two options, the lowest score was chosen to raise awareness and promote discussion around the identified target areas. The most relevant points identified in the evaluation are presented in the Result and Discussion Section.

## Results and discussion

### Evaluation of DANMAP using the OH-EpiCap tool

Given that integrated surveillance has been a pillar of DANMAP for more than 25 years, the program scored highly in all three dimensions identified by OH-EpiCap, with averaging scores above 3 in all target areas. The average evaluation score of DANMAP among all questions that composed each of the target areas covered by the OH-EpiCap can be seen graphically in Figure 2.

Since a perfect level of “OH-ness” in a surveillance activity does not exist (26), changes in the DANMAP program should be carefully evaluated in relation to their added value to the program’s objectives and cost effectiveness (10). Moreover, changes must be at the level at which their impacts can be understood or estimated (9) using some of the recognized metrics to evaluate the benefits of OH (27).

### Dimension 1: organization

Regarding the dimension organization, DANMAP scored ≥3 in each of the following areas: formalization, coverage/transdisciplinary, resource, evaluation and resilience (Figure 2).

DANMAP operates with a clear OH integrated cross-sectoral aim, based on the views of all stakeholders. Still, the program’s leadership and the steering committee do not include all sectors and stakeholders who could potentially be relevant to OH surveillance of AMR, as the environment is not represented. Therefore, other representatives in these governance structures, from the environmental sector and perhaps the livestock industry, could reinforce the OH approach. To safeguard the impartiality of the decisions and discussions, and to maintain an organization that facilitates the needed action(s), the role and operational methods of new additions to the governance structures of the program should be carefully considered before implemented.

DANMAP fully covers Denmark’s territory. Regarding the populations under surveillance, AMU in the human sector is entirely covered and so are the clinical bacterial isolates tested for AMR. On the animal side, AMU is systematically monitored in all food producing and pet animal species. Moreover, AMR is monitored in food producing animals via pathogenic bacterial isolates collected from diseased animals and from caecum samples of healthy broilers, fattening pigs and bovines (calves), randomly selected at the slaughter lines. On the food side, AMR is monitored in bacterial isolates from retail meat consisting of broilers, pork and beef, of nationally and imported origin. AMR in bacteria from food-producing animals and their meat is monitored according to sampling schemes following the European Union legislation for the harmonized monitoring of AMR in zoonotic bacteria (28). Collection of more consistent information about AMU and AMR in companion animals is under development (29).

Previous national research performed around the turn of the century led to the non-inclusion of environmental data into the surveillance program, because no evidence was found to highlight the importance or necessity of such data and adjoined actions. Since then, Danish livestock production has intensified, and human hospital activity has increased. Hereby, the role of wastewater or manure for the spreading of AMR could potentially have increased. The importance of such transmission sources could be further investigated, and if judged as part of relevant exposure pathways, they could be included in the AMR surveillance in a systematic way. Moreover, the consequences of interaction between resistant bacteria originating from the human and animal components with the ubiquitous bacteria in soil could also be considered and investigated, where and if judged relevant. The necessary data, methodologies, and analyses to address this issue are currently being considered. Co-operation with the existing surveillance of diseases/infections in wild game and bi-valved mussels using the samples for analysing bacteria and their resistances might also be a cost-effective way of monitoring presence and potential for spread of AMR in the environment. This is addressed in ongoing DANMAP projects.

Over time, DANMAP has evolved, adapting to new challenges and optimizing content and processes, most of it following internal evaluations or recommendations based on expert opinions. Nonetheless, more regular evaluations could have been performed using a standardized methodology, which could probably have eased a timelier implementation of various proposed corrective measures.

Resources are shared in DANMAP, whenever relevant, but given the multi-institutional nature of the program, this is not always efficient when it comes to sharing equipment or highly trained staff. Appropriate training to manage the tasks at hand is given to the staff involved in the data management and analytical activities. Still, investment in future development and improvement of existing methods and analytical skills should be considered to maintain the high dataflow and adapt to changing methods and datatypes generated.

For the current aims of the program, economic and human resources are considered as sufficient and sustainable, as DANMAP encompasses most of the disciplines that are currently considered relevant to the OH surveillance. The program has successfully and efficiently adapted to previous critical situations, bringing in more expertise whenever needed. To consistently investigate in emerging issues and include them in the surveillance program objectives, an extension of the budget would be required, which would allow more staffing resources for investigation of relevant areas to include. This would also be the case for addition of already considered new components to the surveillance program, e.g., environmental monitoring, the continued expansion of molecular-based surveillance and its integration in the different monitored sectors and the transition to more real-time surveillance than seen at current.

Relevant supporting documentation related to DANMAP, including standard operating procedures, data collection and analytical procedures should preferably be compiled and shared at one common digital point, increasing the public accessibility to the generated results. This should also include a description of governance procedures and stakeholders’ roles and responsibilities. In addition, it should be evaluated whether the program needs more visibility to increase its impact and usefulness for, e.g., antibiotic stewardship programs. This would contribute to the overall transparency of the program including improved applicability of the generated results into mitigating actions.

### Dimension 2: operational activities

Regarding the dimension operational activities, DANMAP scored >3 for each of the areas that formed part of this dimension: data collection/methods, data sharing, data analysis and interpretation, and communication (Figure 2).

The design of surveillance protocols on the animal side is mainly established by the European Union requirements; when new additions to this are being considered, there is an effective collaboration between the sectors involved. As an example, DANMAP 2022 includes for the first time, the results of Extended Spectrum Beta-Lactamase, Ampicillin Class C beta-lactamases -, and Carbapenemase-producing *E. coli* monitoring in turkey meat, as defined in the recently implemented Decision 2020/1729/EU (13). Moreover, the lines of intra- and cross-sectoral communication were improved during the COVID-19 pandemic, which demanded close collaboration, not only within the human health sector, but also with inclusion of the animal production and environmental sector. Regarding data collection protocols, only intra-sectoral collaboration is considered relevant to conduct the current activities of the program.

Laboratory techniques and procedures are coordinated between the responsible actors. Harmonization of indicators for data analysis across sectors and methodology for sampling the animal population for AMR surveillance could possibly be improved. The selection of indicators monitored in animals and humans could, perhaps, be harmonized in a more meaningful way, e.g., *Enterococcus faecalis* is currently monitored in both animals and humans, but only *Enterococcus faecium* is being whole genome sequenced and typed in samples from humans. While both species are jointly responsible for most human infections by enterococci, the proportion of vancomycin resistant invasive isolates has remained stable among *E. faecalis* but increased markedly among *E. faecium* from approximately 2% in 2012 to 14% in 2022. Whole genome sequencing also of *E. faecalis* and *E. faecium* isolates from animal populations would ensure the detection of cross-sectoral transmission. Also, in a truly all-encompassing program, healthy humans and wildlife could be regularly monitored, but this would inevitably come with multiple challenges related to budget and logistics.

When relevant, sectors share data warehouses and digital analytical tools. Joint multi-sectorial analysis could potentially be improved in the future, given that cross-sectoral data sharing agreements would be developed. Frequent and systematic evaluations of data quality are taking place and handled according to the FAIR principles, implying findability, accessibility, interoperability, and reusability.

Scientific expertise is always shared across sectors and upon request, which contributes to the overall transparency and internal/cross-sectoral communication. Data and findings are shared among sectors whenever this is found relevant by the actors. Still, despite the publication of data in the annual report and upon request, communication of findings to the political level could be evaluated and improved for sustained political attention and support. Making data more accessible to the stakeholders by developing dissemination platforms that are fast and easy to consult and understand is on the agenda. Even though not in real-time, the steering committee is informed in a timely manner about the emergence of possible hazards, but translation into action takes time.

### Dimension 3: impact

Regarding the dimension impact, DANMAP scored >3 for each of the areas that form part of this dimension: technical outputs, collaborative added value immediate and intermediate outcomes, and ultimate outcomes (Figure 2).

The program follows a clear national (15) and European OH strategy (30). The steering committee and coordinating actors from the livestock and human sectors are actively involved in the public communication of results, with the annual release of the national report on AMU and AMR and many scientific publications, which frequently involve multi-sectoral national and internationally established collaborations (3, 4). Given the dimension of the entire DANMAP program and the multiple actors it involves, there are no clear figures regarding the full operational costs of the program.

Integrated surveillance has been the foundation of DANMAP from the very beginning. Therefore, questions in OH-EpiCap regarding the added value of adapting to a OH response were not considered relevant in our evaluation. Given the more than 25 years of the program, its impact on epidemiological knowledge of AMR is clear (16). DANMAP has guided sector-specific interventions and policy changes, which have highly contributed to achieve the goals established in the national action plan. One example is the current use of AM in the Danish pig sector, which shows almost no use of 3rd and 4th generation cephalosporines and fluoroquinolones (16). In Denmark, there is a strong will for working collaboratively, which may be one additional explanation for why the OH networks function well in Denmark, and the level of awareness among the stakeholders is very high, even if translation into action could be further improved.

### Using OH-EpiCap tool to evaluate integrated AMR surveillance systems

The OH-EpiCap tool facilitates a quick assessment of certain essential components in OH collaboration that could lead to possible reforms, as has been experienced in Denmark. The tool allowed the actors involved in DANMAP to pinpoint certain components of the program where there is room for improvement to increase the OH-ness of the system. Nevertheless, a more detailed and precise analysis should be conducted to complement the evaluation provided by OH-EpiCap. Some of the identified components have already been previously recognized by program’s management. Still, discussing these issues again, considering recent technological and scientific advancements was considered a positive and valuable experience.

Stakeholders from distinct backgrounds, with diverging perceptions and expectations can be involved in the evaluation processes of a surveillance system using the OH-EpiCap tool. To reduce the possible bias and the overall subjectivity of the evaluation, a consensus among respondents is required to select a final answer (19). The simple and efficient design of the OH-EpiCap makes it a user-friendly addition to the field of existing tools and frameworks to evaluate AMR surveillance (31). However, when assessing complex systems such as DANMAP, in relation to a topic as broad as OH, one should be mindful about the aspects and activities thereof that are not being evaluated (26).

As stated in the Introduction, a full review of OH-EpiCap is presented in a separate paper reporting from nine case studies, all using the CoEvalAMR user’s perspective methodology (22, 32) focusing on the functional aspects and content themes as well as a SWOT-like analysis to provide feedback on the application of the OH-EpiCap (21).

## Conclusion

The process related to evaluating DANMAP using the OH-EpiCap tool provided a good overview of the program’s degree of OH-ness. Moreover, it facilitated the identification of components that could potentially be further improved or included in future reforms, to possibly increase the integrated nature of the program, such as environmental AMR surveillance components Therefore, the authors recognize the value of using OH-EpiCap to initiate an evaluation of the OH-ness of national AMR surveillance programs. Still, as OH-EpiCap is mainly providing an overview, feasibility, requirements and relevance of any additional activities in a program should be considered carefully before implementation. The OH-EpiCap tool not suitable for the technical evaluation of a surveillance program, i.e., sample sizes, matrices etc.

It was concluded that DANMAP demonstrates a high level of adherence to the OH concept, covering relevant aspects for humans and animals, whereas environmental aspects are missing. If it is judged that incorporation of environmental sampling would be valuable, budgetary implications should be foreseen and handled.

## Data availability statement

The original contributions presented in the study are included in the article/supplementary material, further inquiries can be directed to the corresponding author.

## Author contributions

PM drafted the paper with inputs from all authors. LA, MS, and US conducted the evaluation. All authors contributed to the article and approved the submitted version.
